# Retraction: Revamping of chronic respiratory diseases in low- and middle-income countries

**DOI:** 10.3389/fpubh.2023.1207917

**Published:** 2023-05-04

**Authors:** Frontiers Editorial Office

Following concerns regarding the originality of the article, an investigation was conducted in accordance with Frontiers' policies. It was found that the complaint was valid and that the article should be retracted, because of an unacceptable level of similarity to an article published by Meghji J, Mortimer K, Agusti A, Allwood BW, Asher I, Bateman ED, et al. Improving lung health in low-income and middle-income countries: from challenges to solutions. *Lancet*. (2021) 397:10277. doi: 10.1016/S0140-6736(21)00458-X.

This retraction was approved by the Chief Editors of Frontiers in Public Health and the Editor-in-Chief of Frontiers. The authors did not agree to this retraction.

